# Fatty Acid Composition and Thermotropic Behavior of Glycolipids and Other Membrane Lipids of *Ulva lactuca* (Chlorophyta) Inhabiting Different Climatic Zones

**DOI:** 10.3390/md16120494

**Published:** 2018-12-07

**Authors:** Eduard Kostetsky, Natalia Chopenko, Maria Barkina, Peter Velansky, Nina Sanina

**Affiliations:** 1 Department of Biochemistry, Microbiology and Biotechnology, School of Natural Sciences, Far Eastern Federal University, Vladivostok 690091, Russia; kostetskiy.yeya@dvfu.ru (E.K.); natali_1389@mail.ru (N.C.); marybarkin@yandex.ru (M.B.); velanskiy.pv@dvfu.ru (P.V.); 2 National Scientific Center of Marine Biology, Far Eastern Branch of Russian Academy of Sciences, Vladivostok 690041, Russia

**Keywords:** glycolipids, phospholipids, betaine lipid, fatty acids, differential scanning calorimetry, thermal adaptation

## Abstract

Increasing global temperatures are expected to increase the risk of extinction of various species due to acceleration in the pace of shifting climate zones. Nevertheless, there is no information on the physicochemical properties of membrane lipids that enable the adaptation of the algae to different climatic zones. The present work aimed to compare fatty acid composition and thermal transitions of membrane lipids from green macroalgae *Ulva lactuca* harvested in the Sea of Japan and the Adriatic Sea in summer. *U. lactuca* inhabiting the Adriatic Sea had bleached parts of thalli which were completely devoid of chloroplast glycolipids. The adaptation to a warmer climatic zone was also accompanied by a significant decrease in the ratio between unsaturated and saturated fatty acids (UFA/SFA) of membrane lipids, especially in bleached thalli. Hence, bleaching of algae is probably associated with the significant decrease of the UFA/SFA ratio in glycolipids. The decreasing ratio of n-3/n-6 polyunsaturated fatty acids (PUFAs) was observed in extra-plastidial lipids and only in the major glycolipid, non-lamellar monogalactosyldiacylglycerol. The opposite thermotropic behavior of non-lamellar and lamellar glycolipids can contribute to maintenance of the highly dynamic structure of thylakoid membranes of algae in response to the increasing temperatures of climatic zones.

## 1. Introduction

Over the last few decades, the temperature of seawaters has experienced changes that affect the Earth’s ecological state on a global scale. Increasing global temperature is expected to increase the risk of extinction of various species in the future due to acceleration in the pace of shifting climate zones [1]. 

The primary interface between the environment and the organism’s cells are the cell membranes, which are structurally based on the lipid matrix. The maintenance of the liquid crystalline state of the lipid matrix is necessary for the optimal functioning of biological membranes at different changes in the ambient temperature. Therefore, the primary effects of temperature, which is the most powerful environmental factor, are related to the compensatory molecular mechanisms directed to maintain the liquid crystalline state of the membrane lipid matrix [2,3]. This mechanism, which is called homeoviscous adaptation, is mainly realized due to rearrangements in the fatty acid composition of membrane lipids [4,5]. The efficiency of homeoviscous adaptation could be estimated by the lipid thermal transitions from a crystalline (gel) state to a liquid crystalline one. Our earlier studies on the fatty acid composition and phase transitions of the individual polar lipids from taxonomically different marine macrophytes inhabiting the Sea of Japan showed that the decrease in the ratio between unsaturated (UFA) and saturated fatty acids (SFA) was accompanied by the decrease in the ratio between n-3 and n-6 polyunsaturated fatty acids (PUFAs) during acclimatization from winter to summer. Despite the larger changes of these ratios in photosynthetic lipids (glycolipids monogalactosyldiacylglycerol (MGDG), digalactosyldiacylglycerol (DGDG), sulfoquinovosyldiacylglycerol (SQDG) and phospholipid phosphatidyldiacylglycerol (PG)), peak maximum temperatures (T_max_) of their thermal transitions did not increase in contrast to the respective adaptive changes of non-photosynthetic phospholipids (phosphatidylcholine (PC)), (phosphatidylethanolamine) (PE)) and betaine lipid 1,2-diacylglycero-O-4’-(*N*,*N*,*N*-tri-methyl)-homoserine (DGTS) [6].

Extreme temperatures strongly affect the distribution of plants in the world. *Ulva lactuca* (Ulvales; Chlorophyta), widely known as *U. fenestrata*, is the most widespread edible green algae in the seas of all climatic zones. Nevertheless, there is no information on what features in physicochemical properties of membrane lipids facilitate the adaptation of the algae to the conditions of different climatic zones.

Moreover, abnormally high seawater temperatures could result in the partial or even total bleaching thalli of algae due to the loss of chlorophyll and other photosynthetic pigments that is accompanied by growth inhibition of plants [7,8]. However, the underlying mechanism and the role of the lipid matrix in these processes remain unknown.

Therefore, the aim of present work was to clarify the differences between the fatty acid composition and the thermotropic behavior of membrane lipids from *U. lactuca* adjusted to the conditions of different climatic zones – the Adriatic Sea (Mediterranean subtropical climatic zone) and the Sea of Japan (moderate climatic zone) as well as between green and bleached thalli of *U. lactuca* inhabiting the Adriatic Sea. The major membrane lipid of *U. lactuca* and other marine macrophytes MGDG is an important component of tubular immunostimulating complexes (TI-complexes) [9] that affect the conformation and immunogenicity of protein antigens [10,11]. Therefore, the results of the present work are also needed to modulate the lipid surrounding of subunit antigens incorporated in TI-complexes and to enhance their adjuvant effect.

## 2. Results and Discussion

To characterize differences in physicochemical properties of membrane lipids from *U. lactuca* adapted to the conditions of different climatic zones, glycolipids (MGDG, DGDG, SQDG) and the major phospholipids (PG and PE) [12] were isolated from algae harvested in the Adriatic Sea and the Sea of Japan in August, when the average seawater temperatures reach 26 °C and 22 °C, respectively. Betaine lipid DGTS which substitutes PC in green algae [13,14] was also isolated from the algae.

Our earlier study has shown that the percentage of glycolipids, phospholipids and DGTS in *U. lactuca* harvested in the Sea of Japan in summer is 52%, 17% and 15% of total lipids without triacylglycerols, respectively. MGDG is the dominant glycolipid (37% of total lipids without triacylglycerols), whereas the content of DGDG and SQDG is much less (10% and 5% of total lipids without triacylglycerols, respectively). Major phospholipids of *U. lactuca* are PG and PE (6% and 3% of total lipids without triacylglycerols, respectively). Lipid composition of *U. lactuca* is not highly dependent on season [15].

**Figure 1:**
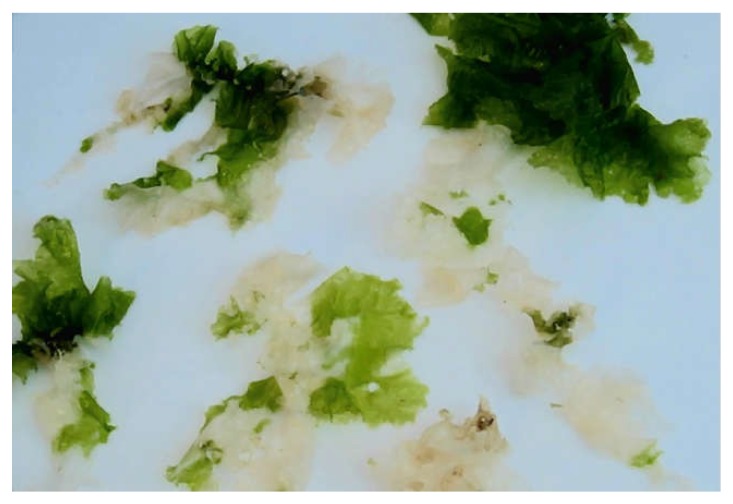
Green and bleached parts of thalli of *Ulva lactuca* harvested in the Adriatic Sea.

*U. lactuca* harvested in the Adriatic Sea had green and bleached parts of thalli (Figure 1). The last ones seem to appear due to the high-temperature stress, because the seawater temperature of 26 °C and higher greatly inhibits the growth of *U. lactuca* [16]. This phenomenon is called thermal bleaching [7] because of the loss of chlorophyll and other pigments. The analysis of lipid composition has shown that bleached parts of the algal thalli were completely devoid of chloroplast-specific glycolipids, which are essential not only for the formation of lipid bilayers of chloroplast membranes, but also for embedding photosynthetic complexes in thylakoid membranes and as integral components of these complexes [17]. It was confirmed earlier that deficient synthesis of chloroplast-specific lipids in Arabidopsis mutants really results in a decrease of chlorophyll content, defects in chloroplast ultrastructure and reduced photosynthetic activity [18].

However, thermal bleaching parts of algae thalli contained extra-plastidial PE and DGTS as well PG, which not only plays a crucial role in the structure and function of photosynthetic complexes in thylakoid membranes but is also found in extra-plastidial membranes [19]. As such, cells of the bleaching parts were completely devoid of chloroplasts but retained extra-plastidial membranes and the ability to adjust them under conditions of heat stress, taking into account the adequate rearrangements in the fatty acid composition of PE, PG and DGTS (Table 1 and Table 2).

### 2.1. The Fatty Acid Composition of Phospholipids

The fatty acid composition of major phospholipids isolated from *U. lactuca* is shown in Table 1. PG and PE from green thalli of algae inhabiting the Sea of Japan and the Adriatic Sea comprised the major fatty acid 16:0 with lesser amounts of 18:3 (n-3) and 18:1 (n-7). The first two fatty acids seem to play the main role in the adaptation of algae to different climatic zones, judging by the significant differences in their content in PG and PE from algae harvested in the Sea of Japan and the Adriatic Sea. However, the most profound changes occurred in bleached parts of algae from the Adriatic Sea. The content of PUFA 18:3 (n-3) was greatly reduced, whereas the level of 16:0 increased in the following line of the PG and PE sources: green algae from the Sea of Japan → green algae from the Adriatic Sea → bleached algae from the Adriatic Sea.

The percentage of 18:1 (n-7) in PE also increased in the same order. Especially pronounced changes occurred in total parameters of the fatty acid composition. The adaptation of *U. lactuca* to the higher temperature environment was accompanied by the essential increasing content of SFAs and monounsaturated fatty acids (MUFAs), as well as by decreasing percentage of PUFAs in PG and PE. Both phospholipids from bleached thalli were characterized by the prevailing content of SFAs and the lowest level of PUFAs and unsaturation index (UI). Simultaneously, the ratio of n-3/n-6 PUFAs consequently decreased, probably because of the increased demand for more potent mediators derived from n-6 PUFAs [20,21,22] in a warmer environment [6]. Then, changes in total parameters of the fatty acid composition observed in phospholipids from *U. lactuca* are mostly similar to earlier data on season acclimatization of marine macrophytes [6].

### 2.2. The Fatty Acid Composition of Betaine Lipid 1,2-diacylglycero-O-4’-(N,N,N-tri-methyl)-homoserine (DGTS)

The fatty acid composition of betaine lipid DGTS (Table 2) was somewhat different from that of phospholipids from *U. lactuca* (Table 1). It comprised the same major fatty acids: 16:0, 18:1 (n-7), 18:3 (n-3). However, the dominant major UFA was 18:4 (n-3), which percentage was much lower in phospholipids. In addition, the percentage of 22:5 (n-3), which was absent in phospholipids, was considerable in DGTS. Similarly, to phospholipids, the level of SFAs (mostly 16:0) increased and the content of PUFAs (mostly 18:4 (n-3) and 18:3 (n-3)) decreased in the line of DGTS sources: green algae from the Sea of Japan → green algae from the Adriatic Sea → bleached algae from the Adriatic Sea. Unlike phospholipids, the level of MUFAs also increased in this line, although the dominant MUFA 18:1 (n-7) did not participate in the adaption process. Dissimilarity in the fatty acid compositions of DGTS and phospholipids was marked in other algae [23].

The mentioned changes were accompanied by a decrease in UI and ratios of UFA/SFA. However, the ratio of n-3/n-6 PUFAs decreased only in the bleached parts of the algae in comparison with green thalli.

### 2.3. The Fatty Acid Composition of Glycolipids

Glycolipids are the main components of photosynthetic membranes and associated with the functioning of the photosynthetic apparatus, whereas phospholipids, except PG, are mainly accumulated in non-photosynthetic membranes and differ from glycolipids by biosynthetic pathway [24]. Nevertheless, trends observed in the fatty acid composition of phospholipids and DGTS (Table 1 and Table 2) also remained in glycolipids from *U. lactuca* (Table 3), which mainly contained the same fatty acids (16:0, 16:1 (n-7), 18:1 (n-9), 18:1 (n-7), 18:2 (n-6), 18:3 (n-3) and 18:4 (n-3)) as well 16:3 (n-3) and 16:4 (n-3) found in phospholipids.

The greatest differences between glycolipids occurred in the composition of major fatty acids. The major fatty acids of MGDG were 16:4 (n-3), 18:4 (n-3), 18:3 (n-3), 18:1 (n-7), while DGDG mainly comprised 18:3 (n-3), 16:0, 18:2 (n-6) and 18:1 (n-7) in order of decreasing percentage. The major fatty acids of SQDG were similar to those of DGDG except for 18:2 (n-6), for which the percentage did not exceed 3.5%.

However, SQDG from *U. lactuca* contained the highest level of *cis*-vaccenic acid 18:1 (n-7), which is not characteristic for other marine macrophytes [6,12]. Also, this glycolipid was the most enriched in SFA 16:0 (more than 50% of total fatty acids) unlike MGDG where the percentage of 16:0 and SFAs as a whole was the lowest.

The adaption of *U. lactuca* to a warmer climatic zone was accompanied by the same changes in total parameters of the fatty acid composition in galactolipids, that was found in phospholipids and DGTS (Table 1 and Table 2): the levels of SFAs and MUFAs increased, whereas other parameters (PUFAs, n-3/n-6 PUFAs, UI and UFA/SFA) decreased. This suggests that bleaching of *U. lactuca* has been accompanied by the strengthening of the trend found not only in extra-plastidial lipids, but also in chloroplast-specific glycolipids. 

In SQDG, total parameters except MUFAs and PUFAs changed in the opposite way. The direction of changes in n-3/n-6 PUFAs of DGDG also did not coincide with the general trend.

**Figure 2:**
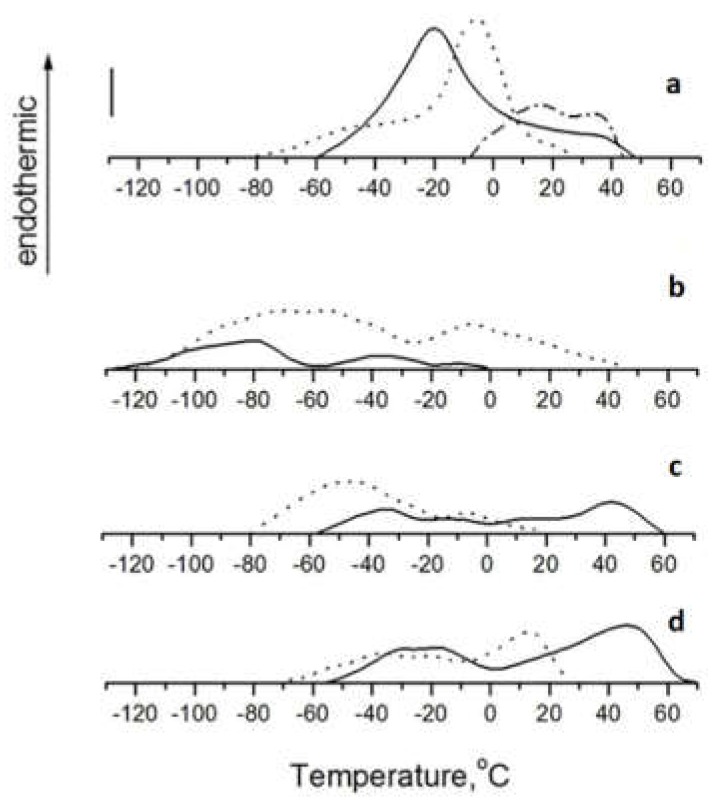
DSC thermograms of betaine lipid DGTS (**a**) and glycolipids MGDG (**b**), DGDG (**c**), SQDG (**d**) from the green thalli of *Ulva lactuca* inhabiting the Sea of Japan (solid curve), the green thalli of algae inhabiting the Adriatic Sea (dotted curve) and the bleached thalli of algae inhabiting the Adriatic Sea (dash-dotted curve). The vertical bar represents 0.5 mW. The scanning rate, 16 °C/min. The sample weight, 10 mg. Each sample was scanned at least three times.

The decline in the ratio of the UFA/SFA was greatest in MGDG, while SQDG had the smallest change. A decrease in the ratio of n-3/n-6 PUFAs was observed in MGDG only. However, this parameter increased or did not change in SQDG and DGDG, respectively. Perhaps the observed dissimilarity in the adaptive changes of the fatty acid composition may be the cause of different thermotropic behavior of glycolipids from *U. lactuca* harvested in the Adriatic Sea and the Sea of Japan (Figure 2).

### 2.4. Thermotropic Behavior and Molecular Species of Polar Lipids

Membrane lipid matrix of ectothermic organisms performs the role of thermosensor. The changes in the physical state of membrane lipids trigger compensatory adjustments in the fatty acid composition of membrane lipids directed to maintain the liquid crystalline state of the membrane lipid matrix, which is optimal for the functioning of living cells [3,25,26]. To assess the efficacy of adaptive rearrangements in the fatty acid composition of major polar lipids from *U. lactuca*, the thermotropic behavior of polar lipids was studied by differential scanning calorimetry (DSC). Calorimetry is the most appropriate method for a detection of the main lipid phase transition from crystalline to liquid crystalline state. Phase transitions of lipids occur due to trans-gauche rotational isomerization of methylene groups about the single C – C bonds along lipid acyl chains. When lipid is heated, the heat flow difference between a sample and a reference is scanned in some temperature range. This endothermic process is visualized by DSC as an integral curve (thermogram) of dependence between a heat capacity and temperature. The peak maximum temperature of thermal transition (T_max_) is characteristic for a lipid sample of a definite chemical structure [27]. Additional peak(s) with lower heat capacity often occurred on thermograms of natural membrane lipids, which is probably due to their phase separation [28,29].

Thermograms and T_max_ of thermal transitions of extra-plastidial betaine lipid DGTS and chloroplast-specific glycolipids MGDG, DGDG and SQDG of *U. lactuca* inhabiting the Sea of Japan and the Adriatic Sea are shown in Figure 2 and Table 4, respectively.

Phase transition of DGTS from *U. lactuca* inhabiting the Sea of Japan was characterized by the cooperative endothermic peak at −21 °С, which was lower by 15 °С than T_max_ of DGTS from *U. lactuca* inhabiting the Adriatic Sea (Figure 2A). Such a classical adaptive change in the thermotropic behavior is usual for non-photosynthetic phospholipids, especially for PC [6], that confirms functional similarity of DGTS and PC in extra-plastidial membranes. The increase in T_max_ was accompanied by the 1.6-fold decrease in the ratio of UFA/SFA despite a little change in UI of the DGTS from *U. lactuca* inhabiting warmer climatic zone. Namely, the partial substitution (by 11.5%) of highly unsaturated molecular forms MUFA/PUFA and PUFA/PUFA for SFA/MUFA and SFA/PUFA (Table 5) contributed to the increase of T_max_.

The further rise in T_max_ and the shift of the temperature range of the phase transition toward higher temperatures occurred at the thermal transition of DGTS from bleached thalli. The respective thermogram was located at the comparatively narrow temperature range between −8°С and 44 °С and characterized by two poorly resolved peaks at 15 °С (T_max_) and 35 °С. The cooperativity of phase transition significantly reduced likely due to the phase separation in this lipid sample. All changes in the thermotropic behavior of DGTS were accompanied by an increase in the share of SFA in its composition (Table 2). 

Thermogram of MGDG from *U. lactuca*, harvested in the Sea of Japan lied in the temperature range between –128 °С and 0 °С. There were three peaks in this thermogram profile: the major peak at −78 °С (T_max_) and additional peaks with lower heat capacity at −36 °С and −10 °С (Figure 2B). MGDG from *U. lactuca* inhabiting the Adriatic Sea revealed the similar thermotropic behavior, while peaks of phase transition occurred at essentially higher temperatures (major peak at about −60 °С and additional one at –6 °С). The high-temperature limit of phase transition was shifted to 44 °С.

Nevertheless, T_max_ of MGDG remained very low. This can be explained by the high content of UFAs (more than 90% of total fatty acids) (Table 3). Most of them are represented by PUFAs (about 80–90% of total fatty acids). In turn, MGDG is the major factor responsible for the functioning of photosynthetic protein complexes. Hence, the high unsaturation probably allows MGDG to adopt different shapes of co-existing membrane protein complexes and stabilize them [5]. Highly unsaturated non-bilayer MGDG can also facilitate membrane deformation and provide the curvature matching of different proteins to the thylakoid membranes [30]. The increase in saturation of MGDG likely decreases the curvature in thylakoid membranes and promotes the crowding out of membrane proteins from the lipid bilayer that may disrupt the architecture and the functioning of thylakoid membranes. Therefore, MGDG plays a crucial role in the structural flexibility of lipid–light-harvesting complex II (LHCII) macro-assemblies. This lipid mediates the dimerization of photosystem II (PSII), as well as the packaging of PSII and photosystem I (PSI) [17,31]. Also, MGDG is structurally involved in the cytochrome b6/f complex [32] and activates the CF0-CF1-ATPase in chloroplasts [33].

From the point of view of homeoviscous adaptation, the shift of T_max_ to the higher temperatures is the adequate compensatory adjustment, which was provided by the classical decrease in the unsaturation of fatty acids in MGDG. The same changes in the fatty acid composition as well as decreasing n-3/n-6 PUFAs of this glycolipid from *U. lactuca* and other marine macrophytes were observed at the season change from winter to summer [6]. As shown in Table 5, the following major molecular species of MGDGs likely contributed to the formation of the major peak on the respective thermograms: 18:3/16:4, 16:4/18:4, as well as 18:1/16:4, 16:3/18:3, 16:2/18:3 and 18:2/16:4 whose sum was about 70%. The positional distribution of fatty acids within MGDG from algae depends on the fatty acid chain length rather than on their unsaturation degree. The preferential occurrence of 18C and 16C fatty acid residues in *sn*-1 and *sn*-2 positions of the glycerol backbone of MGDG, respectively, is typical for green algae [34]. Higher T_max_ of MGDG from algae inhabiting warmer climatic zone was probably due to the two-fold lower content of major molecular species 18:3/16:4.

In spite of the lowering ratio of UFA/SFA, T_max_ of lamellar DGDG decreased, in contrast to non-lamellar MGDG (Figure 2B). An especially remarkable change occurred in the percentage of MUFAs in DGDG (by the 2.5-fold increase) which reduces phase transition temperature more effectively than PUFAs with three or more double bonds [35,36] (Table 3). MUFAs were situated in the following molecular species of DGDG: 14:0/18:1, 16:1/18:1and 16:1/18:3, whose total content was six-fold higher in DGDG from algae inhabiting the Adriatic Sea in comparison with DGDG from algae inhabiting the Sea of Japan (Table 5). The total indicators (MUFA/PUFA and MUFA/MUFA) also demonstrated the major contribution of the DGDG molecular species containing MUFAs to the lowering of T_max_. In turn, the sharp decrease in the level of highly unsaturated molecular species PUFA/PUFA probably does not effectively influence the thermotropic behavior of DGDG.

Adaptive changes in phase transition of SQDG was similar to that of DGDG (Figure 2C,D, respectively) in spite of essentially different polar groups and the fatty acid composition of these glycolipids. As such, a set of major molecular species of SQDG comprises 16:0/18:3, 16:0/18:1, 16:0/16:0, 16:0/18:4 (Table 5), while highly unsaturated molecular species MUFA/PUFA and PUFA/MUFA, whose high content was detected in DGDG, practically absent in SQDG. As a whole, SQDG is the most saturated glycolipid. Therefore, T_max_ of SQDG is the highest in comparison with T_max_ of other glycolipids from *U. lactuca* regardless of the temperature conditions of the habitat of the algae. The shift of T_max_ of SQDG from *U. lactuca* depending on climatic zones was not as sharp as it was found to be for DGDG (about 60 °С against about 90 °С, respectively) (Figure 2C,D, respectively), which was accompanied by a less pronounced increase in the level of MUFAs. The lower T_max_ of SQDG from *U. lactuca* inhabiting warmer climatic zone also correlated with a threefold lower level of the most high-melting molecular species SFA/SFA, as well as remarkably higher UI and UFA/SFA.

Homeoviscous adaptation is the compensatory mechanism that allows cell membranes to maintain the functionally optimal viscosity and, hence, the integrity of organelles at different environmental temperatures [5]. The infringement of the thylakoid membrane integrity initiates the bleaching of algae at elevated temperatures [7]. Our results on the fatty acid composition of PG, PE and DGTS of bleached parts of *U. lactuca* (Table 1, Table 2 and Table 3) allow us to propose that elevated ambient temperature also induces the significant increase of the fatty acid saturation in the major chloroplast glycolipids and the following destruction of thylakoid membranes, the release of glycolipids and chlorophyll [18].

Opposite changes in the thermotropic behavior of non-lamellar MGDG and lamellar DGDG and SCDG probably maintain the highly dynamic structure of the *U. lactuca* thylakoid membranes in response to an increase in temperature of climatic zones. Therefore, a highly dynamic flexibility of the thylakoid structure can be supported by the fine tuning of MGDG/DGDG ratio which influences the reversible transitions between non-lamellar and lamellar phases in thylakoid lipids [37]. As shown earlier [15], the MGDG/DGDG ratio increases at warm-acclimatization of *U. lactuca*. To compensate for the destabilizing effect of the MGDG elevated level at the adaptation of algae to warmer climatic zone, T_max_ of this glycolipid increased. Instead, the lowering of the DGDG share was accompanied by the decrease of its T_max_.

On the other hand, the hydrogen bonds between the DGDG polar heads of adjacent bilayers result in thylakoid membrane stacking. However, electrostatic repulsion between positively charged molecules of SQDG hinders the stacking of thylakoid membranes, which additionally contributes to the dynamic structure of the thylakoid grana [37]. Probably, another way to maintain the highly dynamic structure of the thylakoid membranes is the decrease of the T_max_ and possibly the level [6] of SQDG from *U. lactuca* inhabiting the warmer climate zone.

The pronounced phase separation, which is characteristic for the thermotropic behavior of all glycolipids may provide a different lipid environment for PSI and PSII segregated into stroma and grana thylakoid membranes, respectively [38]. The higher mobility of protein complexes in stroma thylakoids indirectly confirms our assumption [39].

## 3. Materials and Methods 

### 3.1. Plants

Marine macroalgae *U. lactuca* (Chlorophyta: Ulvales) was harvested in the Peter the Great Gulf of the Sea of Japan (moderate climatic zone) in August at seawater temperature of 24 °C, seawater salinity of 34.5‰, at a depth of about 1.5 m, and in the Adriatic Sea near the western coast of the Istrian peninsula (subtropical climatic zone) in August at seawater temperature of 27 °C, seawater salinity of 36‰, at a depth of about 1.5 m. The anatomical and morphological analysis of thalli of the algae was performed with the Axio Imager light microscope (Carl Zeiss, Oberkochen, Germany). In each case the investigated thalli were in a sterile state, without signs of reproduction [40]. Live algal biomass was collected per 500 g from each place. Adriatic algae had bleached parts of thalli, which were separated from green parts of thalli. Freshly harvested algae were thoroughly cleaned to remove epiphytes, small invertebrates and sand particles, and then heated for 2 minutes in boiling H_2_O to inactivate enzymes.

### 3.2. Extraction and Isolation of Lipids

Total lipids were extracted from algae by the method of Bligh and Dyer [41]. Crude glyco- and phospholipids were isolated from total lipid extract by column chromatography on silica gel by elution with acetone, acetone/benzene/acetic acid/water (200:30:3:10, by vol.) and a gradient of chloroform and methanol, respectively [6]. Betaine lipid DGTS was eluted with chloroform/methanol/benzene/28% aqueous ammonia (65:30:10:6, by vol.). Then, these lipids were purified by preparative silica thin-layer chromatography (TLC) using acetone/benzene/acetic acid/water (200:30:3:10, by vol.) or chloroform/methanol/water (65:25:4, by vol.), respectively. The purity of lipids was checked by two-dimensional silica TLC [42,43].

### 3.3. Analysis of the FattyAcid Composition

The fatty acid composition of chromatographically pure lipids was studied by gas-liquid chromatography as described earlier [6]. Esterification of lipids was performed with acetylchloride/methanol (1:20) at 95 °C for 1 h. Fatty acid methyl esters were extracted with *n*-hexane and purified by TLC. Analysis of fatty acid methyl esters was performed by an Agilent 6898 gas chromatograph (Agilent Technologies, Santa Clara, USA), equipped with a flame-ionization detector, a silica capillary column (25 m × 0.25 mm) with Carbowax 20 M. The carrier gas was helium. Individual peaks of fatty acid methyl esters were identified by comparison of GC *R*_t_S with those of authentic standards of fatty acid methyl esters and by equivalent chain length (ECL). Statistical analysis was carried out using the program Microsoft Excel. The results are presented as mean values ± standard error of triplicate determination.

### 3.4. Calorimetry

The thermotropic behavior of chromatographically pure lipids was studied by differential scanning calorimetry as described earlier [6]. Lipids solubilized in chloroform were introduced into standard aluminum pans. Vacuum dried samples of approximately 10 mg were sealed into pans and placed in a DSC-2M differential scanning calorimeter (Biopribor, Puschino, Russia). Samples were either heated or cooled at 16 °C min between −135 °C and 80 °C at a sensitivity of 5 mW. The position of the maximum of heat capacity vs. temperature plot was recorded as the phase transition temperature, T_max_. The temperature range was calibrated by naphthalene, mercury and indium.

### 3.5. Analysis of the Molecular Species Composition

Analytical separation of molecular species of lipids was performed by high-performance liquid chromatography (HPLC) on chromatograph Shimadzu-LC20 with mass-detector LCMS-2010EV (Shimadzu Corp., Duisburg, Germany). It was used Ascentis C18 column (Supelco, Bellefonte, PA, USA), 25 cm × 2.1 mm, 5 µm particle size. The column was thermostated at a temperature of 45 °C. The flow rate was of 0.3 mL/min. Intervals with the constant eluent composition (5 mM aqueous solution of ammonium acetate/methanol/isopropanol, v/v) were following: 0 min—6:92:2, 30 min—6:79:15, 35–38 min—6:69:25 for MGDG, DGDG, SQDG; 0 min—6:92:2, 45 min—6:86:8, 50–65 min—6:54:40 for DGTS. Mass-detector’s options: electrospray ionization, positive ion detection mode for MGDG, DGDG and DGTS, negative ion—for SQDG; nitrogen flow—1.5 L/min; voltage of capillary—4.5 kV for positive ionization, 3.5 kV for negative ionization; temperature of line of desolvation—250 °С; temperature of input interface—280 °С. The content of molecular species was determined by the peak areas on chromatograms of quasimolecular ions corresponding to each molecular species; *sn*-positions of acyls in lipid structure were not defined [44].

Statistical analysis was carried out using the program Microsoft Excel. The results are presented in the form of mean value ± standard error of triplicate determination.

## 4. Conclusions

The comparison of adaptive changes in the fatty acid composition and thermal transitions of polar lipids of *U. lactuca* allowed us to simulate the situation that is expected due to acceleration in the pace of shifting climate zones. Heat stress was shown to induce an intensified decline in the ratio of UFA/SFA in polar lipids of bleached parts of algal thalli, which is an adequate response from the point of view of the theory of homeoviscous adaptation. However, this process has a flip side. A significant increase in the share of SFA in chloroplast-specific non-bilayer MGDG likely decreases the curvature in thylakoid membranes and promotes the crowding out of different membrane proteins, participating in photosynthesis, from the lipid bilayer that may disrupt the architecture and the functioning of thylakoid membranes and chloroplasts as a whole. The opposite changes in the thermotropic behavior of non-lamellar MGDG and lamellar DGDG and SQDG, are probably directed to maintain a highly dynamic structure of thylakoid membranes of *U. lactuca* in response to increasing temperature of climatic zones. Despite the loss of glycolipids and, therefore, chloroplast membranes at heat stress, lipids of extra-plastidial membranes and other likely related functions exhibit thermotolerance, which can only prolong the life of *U. lactuca* and possibly of other algae in conditions of shifting climatic zones due to the increasing global temperature.

## Figures and Tables

**Table 1:** The fatty acid composition of phospholipids from green and bleached thalli of *Ulva lactuca* harvested in the Adriatic Sea and from green thalli of the same algae harvested in the Sea of Japan (% of total fatty acids).

Fatty Acids	Phosphatidylglycerol			Phosphatidylethanolamine		
	The Sea of Japan	The Adriatic Sea		The Sea of Japan	The Adriatic Sea	
	Green Thalli		Bleached Thalli	Green Thalli		Bleached Thalli
16:0	35.5 ± 1.2	45.2 ± 0.8	56.2 ± 1.4	22.0 ± 0.6	29.7 ± 0.7	56.2 ± 1.3
16:1 (n-7)	0.9 ± 0.1	2.6 ± 0.2	2.1 ± 0.1	5.4 ± 0.3	4.4 ± 0.2	2.2 ± 0.1
16:3 (n-3)	8.6 ± 0.3	0.6 ± 0.1	0.2 ± 0.1	0.4 ± 0.1	0.5 ± 0.1	tr.
16:4 (n-3)	2.1 ± 0.2	0.8 ± 0.1	0.4 ± 0.1	0.7 ± 0.1	6.7 ± 0.3	0.9 ± 0.1
18:0	0.3 ± 0.1	0.5 ± 0.1	1.5 ± 0.1	4.6 ± 0.3	2.0 ± 0.1	2.8 ± 0.2
18:1 (n-9)	0.6 ± 0.1	2.3 ± 0.2	2.8 ± 0.2	4.2 ± 0.2	3.4 ± 0.2	4.7 ± 0.2
18:1 (n-7)	17.0 ± 0.7	19.6 ± 0.5	16.9 ± 0.3	10.5 ± 0.8	14.3 ± 0.5	18.7 ± 0.3
18:2 (n-6)	2.3 ± 0.2	2.5 ± 0.1	1.4 ± 0.1	2.7 ± 0.2	5.8 ± 0.4	2.1 ± 0.1
18:3 (n-3)	28.1 ± 0.8	14.1 ± 0.3	3.9 ± 0.1	26.2 ± 0.5	9.7 ± 0.3	2.5 ± 0.1
18:4 (n-3)	1.9 ± 0.1	6.1 ± 0.2	2.0 ± 0.1	1.4 ± 0.1	9.8 ± 0.2	1.5 ± 0.1
22:6 (n-3)	tr.	1.3 ± 0.1	0.9 ± 0.1	2.2 ± 0.1	4.5 ± 0.1	0.3 ± 0.1
SFA	36.4 ± 1.0	46.5 ± 0.7	60.7 ± 0.5	30.0 ± 0.5	34.6 ± 0.5	61.5 ± 0.7
MUFA	19.3 ± 0.4	25.5 ± 0.6	28.0 ± 0.3	24.0 ± 0.4	26.6 ± 0.6	29.2 ± 0.4
PUFA	44.3 ± 0.3	28.0 ± 0.4	11.3 ± 0.3	46.0 ± 0.7	38.8 ± 0.4	9.3 ± 0.3
n-3/n-6 PUFA	16.1 ± 0.3	6.5 ± 0.2	3.1 ± 0.1	9.9 ± 0.2	5.0 ± 0.2	1.9 ± 0.1
UI	152 ± 2	119 ± 1	66 ± 1	173 ± 2	183 ± 2	58 ± 1
UFA/SFA	1.7 ± 0.1	1.1 ± 0.2	0.6 ± 0.1	2.3 ± 0.1	1.9 ± 0.1	0.6 ± 0.1

SFA, UFA, MUFA, PUFA—saturated, unsaturated, monounsaturated, polyunsaturated fatty acids, respectively; UI—unsaturation index. Fatty acids with content below 2% are excluded but considered in calculations of total values. tr.—traces (content less than 0.1%). Mean values ± standard error of triplicate determination.

**Table 2:** The fatty acid composition of betaine lipid 1,2-diacylglycero-O-4´-(*N*,*N*,*N*-tri-methyl)-homoserine (DGTS) from green and bleached thalli of *Ulva lactuca* harvested in the Adriatic Sea and from green thalli of the algae harvested in the Sea of Japan (% of total fatty acids).

Fatty Acids	The Sea of Japan	The Adriatic Sea	
	Green Thalli		Bleached Thalli
14:0	0.4 ± 0.1	1.2 ± 0.1	2.3 ± 0.1
16:0	23.0 ± 0.3	31.0 ± 0.2	38.3 ± 0.3
16:1 (n-9)	0.4 ± 0.1	1.0 ± 0.1	4.3 ± 0.2
16:1 (n-7)	1.5 ± 0.1	5.8 ± 2	5.3 ± 0.2
18:0	0.8 ± 0.1	0.7 ± 0.1	0.7 ± 0.1
18:1 (n-9)	1.8 ± 0.1	1.8 ± 0.1	1.8 ± 0.1
18:1 (n-7)	9.9 ± 0.2	9.8 ± 0.2	9.8 ± 0.2
18:2 (n-6)	7.1 ± 0.2	2.2 ± 0.1	2.2 ± 0.1
18:3 (n-6)	4.5 ± 0.1	2.3 ± 0.1	2.3 ± 0.1
18:3 (n-3)	9.7 ± 0.1	6.6 ± 0.2	6.6 ± 0.2
18:4 (n-3)	28.2 ± 0.1	21.2 ± 0.2	21.2 ± 0.2
20:5 (n-3)	0.6 ± 0.1	3.0 ± 0.1	3.0 ± 0.1
22:5 (n-3)	7.1 ± 0.2	8.6 ± 0.2	8.6 ± 0.2
SFA	24.2 ± 0.2	34.2 ± 0.2	42.6 ± 0.2
MUFA	15.6 ± 0.1	18.0 ± 0.2	25.3 ± 0.2
PUFA	60.2 ± 0.3	47.8 ± 0.1	32.1 ± 0.2
n-3/n-6 PUFA	3.8 ± 0.1	5.8 ± 0.1	2.9 ± 0.1
UI	203 ± 2	195 ± 1	127 ± 1
UFA/SFA	3.1 ± 0.1	1.9 ± 0.1	1.3 ± 0.1

SFA, UFA, MUFA, PUFA—saturated, unsaturated, monounsaturated, polyunsaturated fatty acids, respectively; UI—unsaturation index. Fatty acids with content below 2% are excluded but considered in calculations of total values. Mean values ± standard error of triplicate determination.

**Table 3:** The fatty acid composition of glycolipids from green parts of thalli of *Ulva lactuca* harvested in the Adriatic Sea and the Sea of Japan (% of total fatty acids).

Fatty Acids	MGDG		DGDG		SQDG	
	The Sea of Japan	The Adriatic Sea	The Sea of Japan	The Adriatic Sea	The Sea of Japan	The Adriatic Sea
16:0	2.8 ± 0.1	6.4 ± 0.3	22.2 ± 0.3	28.8 ± 0.3	56.7 ± 0.4	53.4 ± 0.2
16:1 (n-7)	0.8 ± 0.1	2.4 ± 0.2	0.7 ± 0.1	3.9 ± 0.2	1.5 ± 0.1	1.5 ± 0.1
16:2 (n-6)	n.d.	n.d.	7.8 ± 0.2	2.8 ± 0.1	n.d.	n.d.
16:3 (n-3)	3.0 ± 0.2	1.5 ± 0.1	8.3 ± 0.2	2.8 ± 0.1	n.d.	tr.
16:4 (n-3)	37.8 ± 0.4	32.3 ± 0.4	5.6 ± 0.1	1.5 ± 0.1	n.d.	0.3 ± 0.1
18:1 (n-9)	1.0 ± 0.1	2.3 ± 0.1	1.6 ± 0.1	4.4 ± 0.1	0.2 ± 0.1	0.2 ± 0.1
18:1 (n-7)	4.7 ± 0.2	7.0 ± 0.5	5.9 ± 0.2	12.5 ± 0.2	13.4 ± 0.2	22.1 ± 0.3
16:0	2.8 ± 0.1	6.4 ± 0.3	22.2 ± 0.3	28.8 ± 0.3	56.7 ± 0.4	53.4 ± 0.2
18:2 (n-6)	1.5 ± 0.1	1.8 ± 0.1	11.8 ± 0.3	9.4 ± 0.2	3.5 ± 0.1	1.7 ± 0.1
18:3 (n-3)	18.5 ± 0.3	14.5 ± 0.1	30.4 ± 0.5	23.2 ± 0.2	23.6 ± 0.3	11.5 ± 0.2
18:4 (n-3)	25.7 ± 0.4	24.4 ± 0.4	3.5 ± 0.2	3.5 ± 0.1	1.6 ± 0.1	11.2 ± 0.2
18:2 (n-6)	1.5 ± 0.1	1.8 ± 0.1	11.8 ± 0.3	9.4 ± 0.2	3.5 ± 0.1	1.7 ± 0.1
SFA	3.8 ± 0.2	7.0 ± 0.3	23.6 ± 0.4	31.8 ± 0.3	56.7 ± 0.4	53.7 ± 0.3
MUFA	7.9 ± 0.2	13.2 ± 0.3	8.8 ± 0.2	21.6 ± 0.4	15.6 ± 0.3	23.0 ± 0.3
PUFA	88.3 ± 0.8	79.8 ± 0.9	67.6 ± 0.5	46.6 ± 0.7	27.7 ± 0.4	23.3 ± 0.3
n-3/n-6 PUFA	50.7 ± 0.3	35.6 ± 0.4	2.5 ± 0.2	2.7 ± 0.1	7.2 ± 0.2	13.8 ± 0.1
UI	334 ± 3	302 ± 2	203 ± 2	152 ± 1	89 ±1	109 ± 2
UFA/SFA	25.3 ± 0.4	13.3 ± 0.2	3.2 ± 0.1	2.1 ± 0.1	0.8 ± 0.1	0.9 ± 0.1

SFA, UFA, MUFA, PUFA—saturated, unsaturated, monounsaturated, polyunsaturated fatty acids, respectively; UI—unsaturation index. Fatty acids with content below 2% are excluded but considered in calculations of total values. n.d.—not detected, tr.—traces (content less than 0.1%). Mean values ± standard error of triplicate determination.

**Table 4:** Peak maximum temperatures of thermal transitions (T_max_, °C) of betaine lipid DGTS and glycolipids isolated from *Ulva lactuca* inhabiting the Sea of Japan and the Adriatic Sea.

Lipids		T_max_, °C ^1^
DGTS	Green thalli of *U. lactuca* inhabiting the Sea of Japan	−21
	Green thalli of *U. lactuca* inhabiting the Adriatic Sea	−6
	Bleached thalli of *U. lactuca* inhabiting the Adriatic Sea	15
MGDG	Green thalli of *U. lactuca* inhabiting the Sea of Japan	−78
	Green thalli of *U. lactuca* inhabiting the Adriatic Sea	−60
DGDG	Green thalli of *U. lactuca* inhabiting the Sea of Japan	41
	Green thalli of *U. lactuca* inhabiting the Adriatic Sea	−50
SQDG	Green thalli of *U. lactuca* inhabiting the Sea of Japan	50
	Green thalli of *U. lactuca* inhabiting the Adriatic Sea	12

^1^ Standard deviations were less than 1 °C for three replicates.

**Table 5:** The composition of molecular species of betaine lipid DGTS and glycolipids from *Ulva lactuca* inhabiting the Sea of Japan and the Adriatic Sea (% of the sum of molecular species).

Molecular Species	DGTS		MGDG		DGDG		SQDG	
	The Sea of Japan	The Adriatic Sea	The Sea of Japan	The Adriatic Sea	The Sea of Japan	The Adriatic Sea	The Sea of Japan	The Adriatic Sea
16:0/16:0	0.5 ± 0.1	0.3 ± 0.1	n.d.	tr.	0.3 ± 0.1	1.4 ± 0.1	14.2 ± 0.2	4.3 ± 0.2
14:0/18:1	n.d.	n.d.	tr.	0.5 ± 0.1	0.9 ± 0.1	8.3 ± 0.2	2.3 ± 0.1	4.7 ± 0.1
16:0/16:1	3.1 ± 0.1	9.8 ± 0.2	n.d.	n.d.	n.d.	n.d.	n.d.	n.d.
16:0/18:1	1.5 ± 0.1	2.9 ± 0.1	n.d.	0.2 ± 0.1	5.8 ± 0.2	6.7 ± 0.2	23.5 ± 0.3	38.1 ± 0.2
16:0/18:2	6.6 ± 0.2	4.8 ± 0.1	2.3 ± 0.1	1.2 ± 0.1	19.7 ± 0.2	16.2 ± 0.2	6.7 ± 0.2	2.9 ± 0.1
16:0/18:3	15.9 ± 0.1	15.4 ± 0.2	2.3 ± 0.1	3.6 ± 0.2	16.7 ± 0.2	21.1 ± 0.3	46.7 ± 0.2	22.9 ± 0.2
16:0/18:4	13.0 ± 0.1	18.1 ± 0.2	0.5 ± 0.1	1.0 ± 0.1	1.0 ± 0.1	2.9 ± 0.1	3.7 ± 0.1	21.5 ± 0.2
16:0/22:5	4.5 ± 0.2	5.9 ± 0.2	n.d.	n.d.	n.d.	n.d.	tr.	tr.
16:1/18:1	1.2 ± 0.1	1.8 ± 0.1	tr.	0.2 ± 0.1	0.4 ± 0.1	4.6 ± 0.2	tr.	0.3 ± 0.1
16:1/18:3	1.9 ± 0.1	0.6 ± 0.1	2.6 ± 0.2	3.5 ± 0.2	1.9 ± 0.1	6.2 ± 0.1	n.d.	tr.
16:1/16:4	tr.	0.2 ± 0.1	0.7 ± 0.1	3.7 ± 0.2	tr.	tr.	n.d.	n.d.
16:1/18:4	1.6 ± 0.1	3.1 ± 0.1	n.d.	0.8 ± 0.1	n.d.	tr.	n.d.	tr.
18:1/16:3	n.d	0.4 ± 0.1	1.9 ± 0.1	1.5 ± 0.1	2.8 ± 0.2	2.9 ± 0.1	tr.	tr.
18:1/18:3	6.9 ± 0.1	2.7 ± 0.1	0.6 ± 0.1	1.1 ± 0.1	2.3 ± 0.1	3.7 ± 0.2	0.2 ± 0.1	n.d.
18:1/16:4	0.9 ± 0.1	0.4 ± 0.1	7.4 ± 0.2	11.5 ± 0.2	0.6 ± 0.1	1.5 ± 0.1	n.d.	tr.
18:1/18:4	8.6 ± 0.2	6.2 ± 0.2	0.2 ± 0.1	0.9 ± 0.1	0.2 ± 0.1	1.2 ± 0.1	tr.	tr.
16:2/18:3	tr.	n.d.	4.6 ± 0.2	3.0 ± 0.1	10.4 ± 0.2	3.5 ± 0.2	tr.	n.d.
16:3/18:3	0.5 ± 0.1	n.d.	6.5 ± 0.2	4.0 ± 0.2	12.4 ± 0.2	2.1 ± 0.1	tr.	tr.
16:4/18:4	0.4 ± 0.1	0.2 ± 0.1	22.1 ± 0.2	27.8 ± 0.2	3.2 ± 0.1	1.1 ± 0.1	n.d.	n.d.
18:2/16:4	0.2 ± 0.1	n.d.	5.9 ± 0.2	3.6 ± 0.2	0.3 ± 0.1	tr.	n.d.	n.d.
18:3/18:3	1.6 ± 0.1	0.2 ± 0.1	1.6 ± 0.1	1.3 ± 0.1	3.7 ± 0.2	3.5 ± 0.2	tr.	n.d.
18:3/16:4	0.5 ± 0.1	tr.	30.1 ± 0.2	16.7 ± 0.2	5.4 ± 0.1	0.5 ± 0.1	n.d.	n.d.
18:3/18:4	4.3 ± 0.1	0.8 ± 0.1	1.2 ± 0.1	2.6 ± 0.1	0.6 ± 0.1	1.2 ± 0.1	n.d.	n.d.
20:5/22:5	3.8 ± 0.1	4.8 ± 0.1	n.d.	n.d.	n.d.	n.d.	n.d.	n.d.
SFA/SFA	0.5 ± 0.1	0.3 ± 0.1	tr.	tr.	0.9 ± 0.1	1.4 ± 0.1	14.2 ± 0.2	4.9 ± 0.2
SFA/MUFA	4.6 ± 0.1	12.7 ± 0.2	tr.	0.8 ± 0.1	6.8 ± 0.1	16.2 ± 0.2	26.2 ± 0.2	45.5 ± 0.2
SFA/PUFA	50.9 ± 0.2	54.3 ± 0.2	7.6 ± 0.2	7.6 ± 0.2	39.6 ± 0.2	44.1 ± 0.2	58.9 ± 0.2	49.0 ± 0.2
MUFA/MUFA	2.3 ± 0.1	2.7 ± 0.1	tr.	0.2 ± 0.1	0.6 ± 0.1	5.0 ± 0.1	tr.	0.3 ± 0.1
MUFA/PUFA	24.7 ± 0.2	17.9 ± 0.1	15.8 ± 0.3	24.9 ± 0.2	10.3 ± 0.2	18.5 ± 0.1	0.3 ± 0.1	0.3 ± 0.1
PUFA/PUFA	17.0 ± 0.1	12.1 ± 0.1	76.6 ± 0.2	66.5 ± 0.2	41.8 ± 0.2	14.8 ± 0.1	0.4 ± 0.1	tr.

Molecular species with content lower 2% are excluded, but considered in calculations of total values. SFA, MUFA, PUFA—saturated, monounsaturated, polyunsaturated fatty acid, respectively. n.d.—not detected, tr.—traces (content less than 0.1%). Mean values ± standard error of triplicate determination.
